# Why are aid projects less effective in the Pacific?

**DOI:** 10.1111/dpr.12573

**Published:** 2021-09-29

**Authors:** Terence Wood, Sabit Otor, Matthew Dornan

**Affiliations:** ^1^ Research Fellow at the Development Policy Centre Australia; ^2^ Research Associate at the Development Policy Centre Australia; ^3^ Senior Economist at the World Bank Australia

**Keywords:** aid effectiveness, economic geography, the Pacific

## Abstract

**Motivation:**

The Pacific is the world’s most aid‐dependent region, yet available data suggest aid projects are less effective on average in the Pacific than elsewhere in the developing world.

**Purpose:**

This article examines the most likely explanations for lower aid project effectiveness in the Pacific. Explanations include poor governance, restricted levels of political freedom, poor economic performance, isolation, and small populations.

**Methods and approach:**

Three approaches to causal mediation analysis are used to identify which explanatory variables best explain why aid projects are less effective in the Pacific. Aid project effectiveness data come from a multi‐donor dataset of individual aid projects. Data on potential explanatory variables comes from a range of international datasets.

**Findings:**

All three causal mediation approaches point to the isolation of many Pacific countries, alongside comparatively small populations, as being the main impediments to project effectiveness. These findings hold even with a suite of project traits being controlled for and within an analysis in which all the key country variables of interest are controlled for.

**Policy implications:**

Project effectiveness in the Pacific appears to be primarily constrained by variables that cannot themselves be shifted (the region’s countries cannot readily be made less remote or more populous). Improved project effectiveness in the Pacific will require donor practice to carefully adapt to the region’s context. A structured process of donor learning will be needed.

## 1 INTRODUCTION

In recent decades, development progress has been slow in much of the Pacific. Most Pacific Island countries performed poorly against Millennium Development Goals and subsequent progress against the Sustainable Development Goals has been uneven (Pacific Islands Forum Secretariat, 2015, 2018). More so than in any other region on Earth, aid is a central feature of the development landscape in the Pacific. Of the world’s 20 most aid‐dependent countries, 10 are from the region. All of the Pacific countries eligible for official development assistance (ODA) are more aid dependent than the median aid recipient globally (OECD, n.d.).^1^ Aid matters to the Pacific. Yet existing analysis of aid project data strongly suggests aid projects are less effective in the Pacific than elsewhere in the developing world (Feeny & Vuong, 2017; Wood et al., 2020).

In this article, we engage in a detailed empirical attempt at explaining why aid projects are less effective in the Pacific. To do this, we use a large, purpose‐built dataset of aid project appraisals. In the first part of our analysis, we use causal mediation analysis to study which country traits serve as likely explanators of why aid projects are less effective in the region. The findings from this analysis suggest that the remoteness and small populations of many Pacific countries, rather than other potential candidates such as poor governance, are the main reason why aid project effectiveness is lower in the Pacific.

In the second part of our analysis we study which project traits, including size, duration, and sector, have differing effects in the Pacific compared to the rest of the developing world. As we do this, we find no evidence that the effects of project size and duration on project effectiveness differ in the Pacific from other developing countries. When we study project effectiveness across sectors, however, we find evidence that humanitarian projects are notably less effective in the Pacific than they are elsewhere.

This article contributes to the broader literature by being one of the first ever studies to analyse the effectiveness of aid projects in the Pacific—the world’s most aid‐dependent region. The article also demonstrates two potential approaches that future researchers can use to learn more about why aid project effectiveness differs between regions and countries. In our work we also use a large multi‐donor dataset that covers donors and projects not previously included in work on aid project effectiveness. In addition, the article presents two key findings for policy‐makers: first, because the two most likely country‐level constraints on project effectiveness in the Pacific—size and isolation—are aspects of the region’s countries that cannot be readily changed, it is donor practice that will need to adapt; second, the rising impacts of climate change in the Pacific, combined with many Pacific countries’ pre‐existing vulnerability to natural disasters, makes underperformance in humanitarian emergency response an aspect of aid practice in urgent need of donor attention.

The article proceeds as follows. We review relevant literature before explaining our data and methods. Then we present results—starting with why aid projects are less effective in the Pacific, before moving to which types of project traits influence projects in different ways in the Pacific. Finally, we conclude with discussions of the substantive importance of our findings and what they mean for aid policy. We also provide suggestions for future research and research approaches.

## 2 LITERATURE

### 2.1 Quantitative analysis of aid project effectiveness

The quality and impacts of aid can be studied through a wide variety of approaches. For example, principles of good aid practice can be taken from the international agreements such as the Paris declaration, or from academic studies, and donor practice can be evaluated against these principles. (For a lucid discussion of the potential and challenges of this approach see McKee et al., 2020). Case studies and impact evaluations can be used to study individual projects, and at times provide broader insights into the effects of particular types of aid (for example, Banerjee et al., 2015). Cross‐country regressions can offer insights into the relationship between aid flows and country‐level progress in areas such as economic development, human development, and governance. (For examples, including examples from the Pacific, see: Arndt et al., 2015; Feeny, 2005; Feeny & McGillivray, 2010; Galiani et al., 2017; Jones & Tarp, 2016; Pavlov & Sugden, 2006; Wright, 2010).

Alongside work in these and other areas, a small but growing body of research has sought to derive insights into factors contributing to the effectiveness of aid projects through the quantitative analysis of data taken from donors’ project appraisals. This work differs from other approaches in that, unlike case studies and impact evaluations, its focus is broad—it looks at all projects for which there are data, rather than focusing on a single intervention, or type of intervention. Yet at the same time, because it is project oriented, its focus is narrower than assessments of donor practice or econometric investigations into the relationship between aid and development.

The systematic study of aid project appraisals has limitations. Donor institutional incentives may cause appraisals to be overly positive on average. Conversely, projects may deliver indirect benefits that differ from initial objectives, and these may be overlooked in appraisals. Project appraisals are also unlikely to capture positive or negative spillovers from aid. Such limitations mean that the systematic study of project appraisals cannot capture all that matters about aid. The study of project appraisals cannot, for example, answer high‐level questions such as whether aid promotes economic growth, or which donors deliver the best quality aid. Yet project appraisals can still usefully contribute to the understanding of aid work. In particular, their study can provide insights into which types of projects are most likely to succeed and in which circumstances. Accordingly, it is these types of questions that the analysis of aid project appraisals has primarily attempted to tackle.

Until recently, a major factor limiting the systematic study of aid project appraisals has been the availability of data in a form amenable to quantitative analysis. For many years the World Bank was the only donor to make this type of data available (World Bank, n.d.). In 2017, the Asian Development Bank (ADB) added to available material when it released similar data (Asian Development Bank, n.d.). The store of available data was further increased when Professor Dan Honig released a dataset created for his book *Navigation by judgement* (Honig, 2018). In addition to World Bank and ADB data, the Honig dataset included information on six other donors.

Existing analysis of aid project effectiveness data has focused on two types of project traits: those associated with individual aid projects and those associated with the countries projects are run in. The work itself has involved regressions, with data almost always pooled and treated as if it is cross‐sectional, and in which the key dependent variable—project effectiveness as appraised by project reviewers—is treated as binary, ordinal, or continuous (for example, Bulman et al., 2017; Denizer et al., 2013; Feeny & Vuong, 2017; Honig, 2018). Standard project traits included in analysis are project size, duration, and sector. A fair conclusion would be that where these traits have been studied, findings have been mixed. No clear consensus has emerged, for example, that certain sectors are more likely to succeed (Bulman et al., 2017; Denizer et al., 2013; Feeny & Vuong, 2017; Wood et al., 2020). At least two studies have found that projects that were longer in duration were less favourably appraised on average, although other studies have failed to find a relationship (Denizer et al., 2013; Feeny & Vuong, 2017; Wood et al., 2020). Similarly, one influential study of World Bank projects found larger projects to be less successful, yet the opposite finding emerged from analysis of Australian data (Denizer et al., 2013; Wood et al., 2020).

Study of country‐level factors has tended to produce clearer findings. Economic growth is often found to be positively associated with project effectiveness. And, although the relationship is more ambiguous, levels of recipient gross domestic product (GDP) have also been found to be associated with success in some papers (Bulman et al., 2017; Denizer et al., 2013; Feeny & Vuong, 2017; Kilby, 2000; Wood et al., 2020). Generally, when it has been studied, better governance has been found to be positively associated with project effectiveness. However, the relationship between political and civil freedoms, and success, is more mixed. Some studies have found a positive relationship, others have found no relationship or even a negative relationship. Negative relationships have tended to be most pronounced in studies focused on the Asia‐Pacific region (Bulman et al., 2017; Denizer et al., 2013; Feeny & Vuong, 2017; Feil, 2021; Honig et al., 2019; Isham et al., 1997).

### 2.2 Project effectiveness in the Pacific

Three recent studies that focused on aid project effectiveness have specifically looked at the effectiveness of aid projects in the Pacific. All three studies have found that projects in the Pacific are less effective on average than projects in the rest of the developing world (Feeny & Vuong, 2017; Wood & Otor, 2019; Wood et al., 2020). This conclusion from quantitative research about lower aid project effectiveness in the Pacific appears to fit with the qualitative beliefs of at least some aid workers based on their practical experience (for example, Hunt, 2020). Also, a similar finding emerges from analysis of Australian Government Aid Program country‐level data assessing the extent to which country objectives have been met (Howes et al., 2020). While the finding that aid is less effective in the Pacific emerges from a range of sources, no existing work has sought to empirically examine why it exists.

## 3 DATA AND METHODS

The data for the primary outcome in our study—project effectiveness—all come from aid project assessments. Not all donors provide a numeric value to represent the effectiveness of their projects. And not all donors that do, make these values public. However, data are now in the public domain for the following donors: the Australian Government Aid Program; the World Bank; the ADB; the UK’s Department for International Development (DFID) (now part of the Foreign, Commonwealth & Development Office); Deutsche Gesellschaft für Internationale Zusammenarbeit (GIZ), the German government’s development agency; KfW, the German government’s development bank; the International Fund for Agricultural Development (IFAD), a specialized agency of the United Nations; Japan International Cooperation Agency (JICA), the Japanese government aid program; and The Global Fund to Fight AIDS, Tuberculosis and Malaria (GFATM). The data are global, coming from throughout the developing world, including projects in the Pacific as well as many projects from other regions.

Following standard practice, Pacific countries are defined in our analysis as aid‐recipient islands situated in the Pacific Ocean. Reflecting our definition and those countries for which there are available data, the Pacific countries used in our analysis are: Cook Islands, Federated States of Micronesia, Fiji, Kiribati, Marshall Islands, Nauru, Palau, Papua New Guinea, Samoa, Solomon Islands, Tonga, Tuvalu, and Vanuatu.

Project effectiveness is taken from donor reviews and measures the extent to which projects were effective in meeting their original goals. In the data we work with, following Honig (2018) we use effectiveness scores standardized to a six‐point scale (with one being the worst possible score and six the best). For a full discussion of effectiveness scores and their distribution among different donors, see Wood et al. (2020). An obvious concern with using data from donors’ assessments of aid projects is the potential subjectivity of these assessments. Many donors have internal processes in place for double‐checking project appraisals (for example, final aid quality assessments are independently double‐checked in the Australian Government Aid Program, and sent for revision if they are deemed inaccurate). Such procedures may serve as some check on any potential impulse staff may feel to inflate project scores. Importantly, studies that have compared internal appraisal scores with those from independent external evaluators have tended to find little evidence of inflated appraisal scores (Denizer et al., 2013). Most importantly, however, the analytical leverage in our work does not come from absolute appraisal scores, and therefore is free of an obvious risk—that donors and evaluators are too generous in appraising aid projects. Rather, leverage stems from the relative differences in appraisal scores (scores being lower in the Pacific, for example, than they are elsewhere). Unless there is a reason to think some subjective bias shapes relative differences, inference involving them is still valid.

For work that involves more than one donor, one plausible source of bias stems from differences between donors: some donors may be more lenient towards their projects than others. These may also be donors that do less work in certain regions or certain sectors, in which case inferences will be biased. Another potential source of bias is that donor lenience in appraisals may change over time, which would also be an issue if donors simultaneously changed focus over the same time period. Fortunately, these are issues that can be largely accounted for by including donor and project completion date fixed effects in regression models, a method we apply (Bulman et al., 2017; Honig, 2018).

In our work we gathered data on World Bank and ADB projects directly from the organizations’ websites. Other donors, with the exception of Australia, were sourced from the dataset compiled by Honig (2018). In the case of Australian data, we worked with the Australian Government Aid Programme and made use of aid programme reports to build a dataset of project effectiveness scores.^2^ Donor project assessment data is usually accompanied by information on some project specifics such as size, duration, and sector. Where it was not, in some instances we were able to match project assessment data with project specifics from other donor sources. It was not possible to gather data on a large suite of project specifics, a point we return to in discussion. However, we were able to gather data on a core set of important project traits.

In our final dataset we complemented project‐level data with data on the recipient countries the projects were delivered in. These country traits were selected either because they had been shown to influence effectiveness in previous work on aid projects or because existing research on the broader constraints to development in the Pacific suggested the traits could be of importance. (The two traits in the latter category were small populations and remoteness; see Winters & Martins, 2004; World Bank, 2017). We used World Development Indicator data on recipient economic and demographic indicators, World Bank government effectiveness data, Freedom House data on political and civil freedoms (hereafter referred to as “freedom”), and CEPII data for remoteness. CEPII data are standard in trade analysis. They measure the distance between the largest cities in two countries, with distance being weighted by the size of each city vis à vis each country’s total population (Mayer & Zignago, 2011). Following standard practice, we turned these data into a single value for every country in each year the data covered. This value was calculated as the mean distance of each country from every other country on Earth, with country distances weighted by the size of country economies. Once again, this is a standard measure (Bacchetta et al., 2010). The dataset and approach are often used in analysis of remoteness and the Pacific (for example, Horscroft, 2014; World Bank, 2017).

For each country‐level variable of interest, we obtained the value of the variable at the start of each aid project and also an average across project lifespans. (For example, if a project ran in Fiji from 2000 to 2005, for GDP per capita, we used Fiji’s GDP per capita in 2000 and also calculated mean GDP per capita in Fiji from 2000 to 2005). In our analysis, we used the variable from the start of the aid project if there was any risk of reverse causality (the effectiveness of aid projects influencing the variable), otherwise (for variables such as remoteness) we took the average value from across the lifespan of the project.^3^

Although some aid project effectiveness data from the World Bank are available as far back as the 1960s, our analysis was restricted to projects that were assessed from 1996 onwards owing to unavailability of key country‐level variables from earlier periods. This is unproblematic as our interest is in contemporary issues of aid effectiveness.^4^ For the sake of consistency, in all our work—whether bivariate or including multiple controls—we restricted analysis to the same time periods and only to observations for which all variables were present. Table 1 provides basic summary statistics for our data. Table 2 shows the total number of analysed projects by donor.

**TABLE 1:** Summary statistics

Variable	Mean	Std Dev	Min	Max
Project effectiveness rating (1–6)	4.25	1.06	1.00	6.00
Real GDP per capita growth at start of project	3.64	6.20	−34.90	92.12
GDP per capita at start of project (ln)	8.22	0.86	6.13	10.76
Remoteness (000kms) (average over project)	8.75	1.52	5.68	12.68
Population (ln) (average over project)	17.13	2.01	9.15	21.04
Governance (start of project)	−0.52	0.51	−1.90	1.36
Freedom (start of project)	7.81	3.15	2.00	14.00
In Pacific	0.03	0.18	0.00	1.00
Total number of projects	8062			
Number of recipient countries	148			

Notes

All projects assessed are from 1996 or more recent. “In Pacific” is a dummy variable coded 1 if the country is an aid recipient in the Pacific region. The Pacific countries in our sample are: Cook Islands, Federated States of Micronesia, Fiji, Kiribati, Marshall Islands, Nauru, Palau, Papua New Guinea, Samoa, Solomon Islands, Tonga, Tuvalu, and Vanuatu.

**TABLE 2:** Project breakdown by donor

Donor	Projects
Australian Government Aid Program (Australia)	429
Asian Development Bank	751
Department for International Development (UK)	1,676
The Global Fund to Fight AIDS, Tuberculosis and Malaria	101
GIZ (German government development agency)	109
KfW (German government development bank)	342
International Fund for Agricultural Development	25
Japan International Cooperation Agency	501
World Bank	4,128

Notes

All data are from Honig (2019), except data for the World Bank and ADB, which are from those organizations’ websites, and Australia, which were provided to the authors in 2019.

We undertook the first component of our analysis—study of which country traits might explain why the appraised effectiveness of aid projects is lower in the Pacific—using causal mediation analysis. This is a standard approach for estimating the extent to which the effect of one variable on another is mediated by other variables (Imai et al., 2010). In this case, we sought to estimate the extent to which the Pacific effect on project effectiveness was mediated by each of the set of variables detailed in Table 1. These potential mediators were chosen either because they have been shown to impact project effectiveness in existing studies, or—as is the case with remoteness and population—there is good cause to suspect they may be a constraint on project effectiveness in the Pacific because of their broader impacts on the region.

We used three different approaches to causal mediation analysis. First, because of the simple intuitive nature of the approach, we combined analysis in which we studied whether mediators of interest varied systematically between the Pacific and elsewhere, with an iterative set of tests. The iterative tests assessed the cumulative impact of each potential mediator in a regression model in which aid effectiveness was the dependent variable and in which the impact of the Pacific was controlled for. In each iteration of the model, additional potential mediators were added. A sense of the extent to which each potential mediator explained why aid was less effective in the Pacific was provided by the change in the Pacific coefficient as the mediator was added. Formally, this approach is referred to as the “difference method” (VanderWeele, 2016).

The regression equation used in the most basic form of the model was:

(1) $$effectiveness_{i}=\alpha +\beta^{\prime }pacific_{i}+\delta^{\prime }Z_{i}+u_{i}$$

In the model, projects’ effectiveness scores were the dependent variable, ∝ the intercept, β the extent to which project effectiveness differ between the Pacific and elsewhere, Z a vector of controls including all project traits, and donor and completion year fixed effects, and u the error term.

In subsequent iterations the model took the following form:

(2) $${\mathrm{effectiveness}}_{i}=\alpha +\beta^{\prime }\;{\mathrm{pacific}}_{i}+\gamma^{\prime }\;{\mathrm{mediators}}_{i}+{\delta^{\prime }Z}_{i}+u_{i}$$

In which *mediators* was a vector of mediating country traits of interest. With each iteration an additional potential mediator was added to the vector of country traits. As each mediator was added the change in the Pacific dummy was the key point of interest. Country traits which shifted the Pacific dummy’s coefficient substantially when added to Model 2 were likely mediators (Baron & Kenny, 1986; Preacher & Hayes, 2008; VanderWeele, 2016). The regression models used in this approach were ordinary least squares (OLS).

Our second approach was more systematic. In it, we used Seemingly Unrelated Regressions (SUR) to combine estimates of the variation in potential mediating variables between the Pacific and elsewhere with estimates of the variables’ impact on the effect of the Pacific on aid effectiveness. This approach involved simultaneously estimating the following models:

(3) $$mediator_{\mathrm{ij}}=\alpha +\beta^{\prime }\;pacific_{i}+\delta^{\prime }Z_{i}+u_{i}$$

(4) $$effectiveness_{i}=\alpha +\beta^{\prime }\;pacific_{i}+\gamma^{\prime }\;mediators_{i}+\delta^{\prime }Z_{i}+u_{i}$$

Model 3 was estimated one time for each of the *j* mediators of interest and as part of the same series of equations as Model 4. Using SUR allowed us to precisely estimate how much of the Pacific’s effect on aid project effectiveness was mediated through each potential mediator. It also allowed us to consistently estimate standard errors and measures of the statistical significance of each mediator’s impact on the Pacific effect (Baron & Kenny, 1986; Statistical Consulting Group, n.d.; VanderWeele, 2016). Once again, OLS regressions were used.

Our third approach, which we report on in the online appendix, involved using the Karlson, Holm, and Breen (KHB) method, a new means of testing for causal mediation (Karlson & Holm, 2011; Kohler et al., 2011). The KHB approach serves as a useful robustness test. It can be used with clustered standard errors and it can be used in models in which the dependent variable is not treated as continuous (Kohler et al., 2011; Smith et al., 2019). As we show, results from the KHB approach (with clustered standard errors and the dependent variable, project effectiveness, treated as ordinal) were effectively identical to the results presented in the main body of the text. As a further robustness test, in the online appendix we also tested whether results changed substantively in more parsimonious models that included only key country traits and which, in one case, excluded project‐level controls. When we did this, we found no evidence of significantly different findings.

In the second component of our analysis, we used OLS regressions with interaction terms in the models to study which project traits had differing impacts in the Pacific from the rest of the developing world. The approach is shown in equation form in Model 5.

(5) $$effectivenessrate_{i}=\alpha_{0}+\beta^{\prime }pacific_{i}+\gamma^{\prime }\;projecttrait_{\mathrm{ij}}+\beta^{\prime }\;pacific_{i}*\gamma^{\prime }\;projecttrait_{\mathrm{ij}}+\delta^{\prime }Z_{i}+u_{i}$$

Here the dependent variable is the effectiveness score of the project in question, α the intercept, β the Pacific effect and γ the individual suite of *j* project traits on effectiveness. Z is a vector of controls including country traits, and donor and completion year fixed effects. u is the error term. The key variable of interest is the interaction between each of the project traits and the Pacific. The studied project traits were sector, size, and duration.

## 4 RESULTS

**FIGURE 1:**
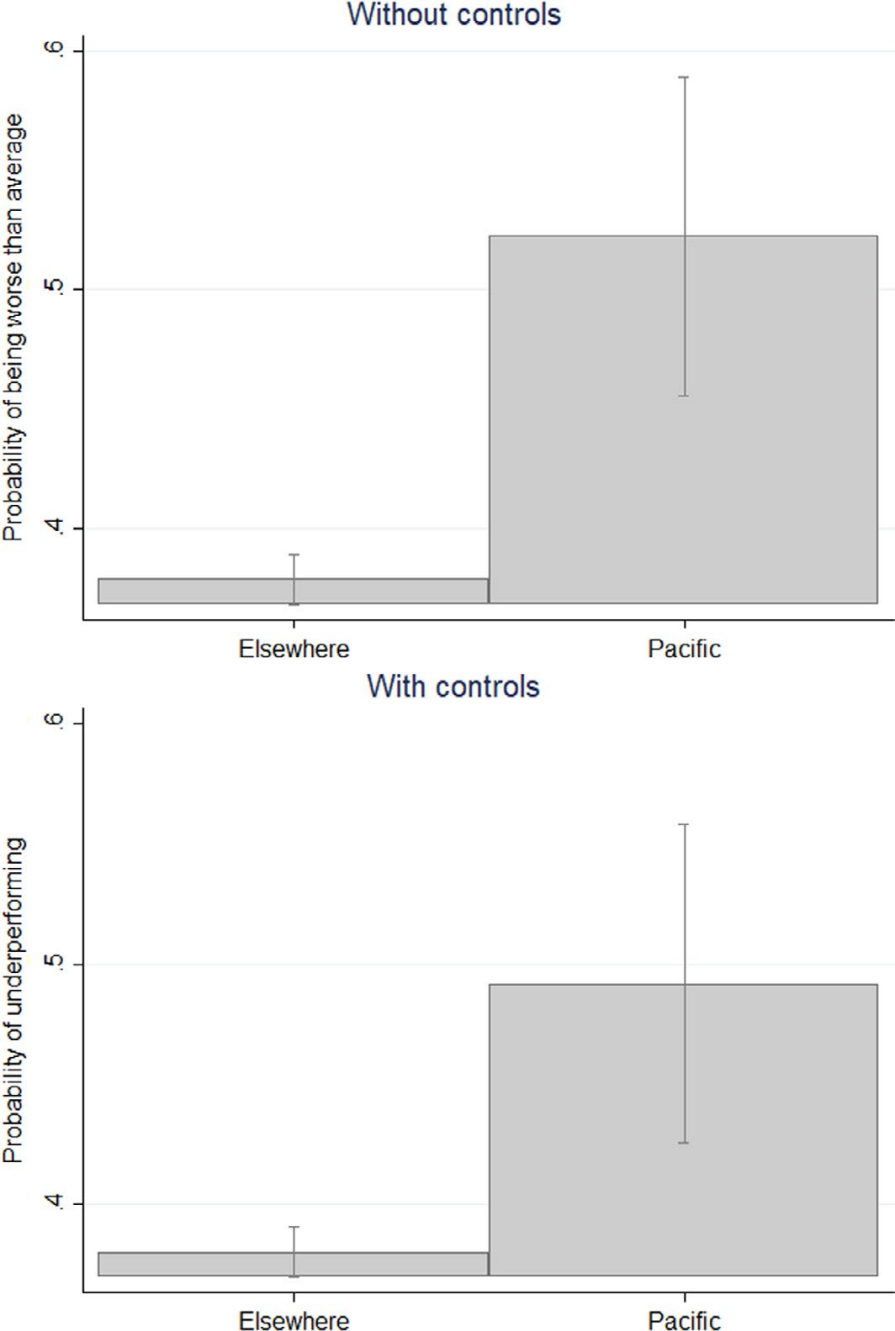
Probability of underperforming, Pacific projects and elsewhere *Notes*: data come from 1996 and thereafter. Data are from all donors with projects in the Pacific. Values are predicted probabilities of projects performing worse than the donor’s mean project in that year. Predicted probabilities come from logistic regressions. In the second panel, regressions are run with project traits controlled for.

Figure 1 compares project effectiveness between the Pacific and the rest of the developing world. As we noted above, different aid donors may be more or less lenient in assessing their projects, and donors may become more or less lenient over time. As stated, in our formal analysis we account for this issue using donor and completion year fixed effects. However, to offer a simple visual means of demonstrating difference in project effectiveness, which accounts for these issues, we generated a binary variable that indicated whether a project had performed below its donor’s average in the year in which it was assessed. The relationship between this binary and the Pacific was then estimated using a logistic regression. The resulting average probability that a project will be below donor average is shown for the Pacific and elsewhere in Figure 1. The first panel in the regression is a simple comparison, the second panel comes from a regression model in which project traits such as size and sector are controlled for.

The predicted probability of underperformance in the Pacific is more than 10 percentage points higher in both panels. Projects in the Pacific are certainly not guaranteed to fail, but they are much more likely to underperform than projects in the rest of the developing world.^5^

In Table 3, we compare whether the variables of interest differ on average between the Pacific region and elsewhere. The values in the table show the mean score for each variable averaged across projects. Averages are provided for projects run outside of the Pacific and projects inside the Pacific. Averages are compared in the “Difference” column.

**TABLE 3:** Key variables in the Pacific and elsewhere

	Non‐Pacific mean	Pacific mean	Difference
Governance	−0.532	−0.141	0.391***
	(0.006)	(0.026)	
Freedom	7.710	10.707	2.998***
	(0.035)	(0.129)	
Growth	3.719	1.261	−2.458***
	(0.071)	(0.267)	
GDP Per capita (ln)	8.222	8.139	−0.083
	(0.010)	(0.027)	
Remoteness	8.657	11.566	2.909***
	(0.016)	(0.034)	
Population (ln)	17.274	13.020	−4.254***
	(0.021)	(0.115)	

Standard errors in parentheses; P‐values from t‐test of means; *p < 0.10, **p < 0.05, ***p < 0.01.

The table shows that, on average, the countries of the Pacific are better governed and freer than the rest of the developing world (at least as captured in standard measures). Economic growth is lower in Pacific countries. GDP per capita is also lower if anything, although the difference is not statistically significant. As would be expected, Pacific countries are more remote on average and have smaller populations.

Table 4 presents the results of a series of regressions in which aid project effectiveness is the dependent variable and projects are the unit of analysis. In each of these regressions, project traits are controlled for and donor and completion year fixed effects added. The first independent variable in each regression is a dummy variable for the Pacific. It represents the “Pacific effect”: the extent to which project effectiveness differs between the Pacific and elsewhere. The first regression contains the Pacific (alongside project controls and fixed effects) as the sole independent variable. In each subsequent regression, potential mediating variables are added one at a time.

**TABLE 4:** Aid project effectiveness, the Pacific dummy, and added variables

	Pacific	Governance	Freedom	Growth	GDP	Remote	Pop
Pacific	−0.15**	−0.23***	−0.18***	−0.16**	−0.14**	−0.07	0.02
	(0.06)	(0.06)	(0.06)	(0.06)	(0.06)	(0.07)	(0.07)
Governance (initial)		0.14***	0.31***	0.30***	0.27***	0.28***	0.29***
		(0.03)	(0.04)	(0.04)	(0.04)	(0.04)	(0.04)
Freedom (initial)			−0.04***	−0.04***	−0.04***	−0.03***	−0.03***
			(0.01)	(0.01)	(0.01)	(0.01)	(0.01)
Growth (initial)				0.01**	0.01**	0.01**	0.01**
				(0.00)	(0.00)	(0.00)	(0.00)
GDP per capita (initial; ln)					0.04**	0.03	0.02
					(0.02)	(0.02)	(0.02)
Remoteness						−0.03***	−0.04***
						(0.01)	(0.01)
Population							0.02***
							(0.01)
Donor FE	Yes	Yes	Yes	Yes	Yes	Yes	Yes
Completion FE	Yes	Yes	Yes	Yes	Yes	Yes	Yes
Sector FE	Yes	Yes	Yes	Yes	Yes	Yes	Yes
Size control	Yes	Yes	Yes	Yes	Yes	Yes	Yes
Duration control	Yes	Yes	Yes	Yes	Yes	Yes	Yes
R2	0.12	0.13	0.13	0.14	0.14	0.14	0.14
N	8062	8062	8062	8062	8062	8062	8062

Notes

Estimates come from OLS regressions with clustered standard errors in parentheses. The unit of analysis is the individual project. The dependent variable is the project effectiveness score. Project traits are controlled for in all models. Donor and completion year fixed effects are included in all models. *p < 0.10, **p < 0.05, ***p < 0.01.

It is instructive, as these variables are added, to examine the change in the coefficient for the Pacific dummy. Any variable that clearly shifts the coefficient for the Pacific dummy is a likely mediator.

First, when governance is added, the Pacific coefficient actually becomes larger (that is, its difference from zero becomes greater). This suggests governance is a moderating variable: because good governance boosts aid project effectiveness, and because governance is better in the Pacific, the finding indicates the negative effect of the Pacific on project effectiveness would actually be greater were it not for the positive influence of comparatively good governance. Adding the freedom variable reduces the magnitude of the Pacific effect considerably. Growth and GDP also reduce the magnitude but their impact is small. Remoteness, on the other hand, has a substantial impact, and for the first time the coefficient of the Pacific’s effect on project effectiveness ceases to be statistically significant. When population is included, the coefficient for the Pacific changes substantially again, actually becoming positive albeit not statistically significantly different from zero.

The fact the Pacific coefficient is effectively zero at the end of the analysis suggests the negative effect of the Pacific on project effectiveness is completely mediated by these variables. The coefficients for all the variables except GDP per capita are statistically significant in the final model, implying that all variables play a role of some sort in explaining the Pacific effect.

In Table 5 we build upon our initial findings by reporting on the results of more complex analysis in which SUR were employed.

**TABLE 5:** Results of main mediation analysis

Total effect	−0.150		
Mediated effect	−0.166		
Remaining direct effect	0.016		

Mediator	Effect	Std Err	p‐value
Governance	0.15	0.02	0.00
Freedom	−0.11	0.02	0.00
Growth	−0.02	0.01	0.00
GDP (ln)	0.00	0.00	0.25
Remoteness	−0.10	0.02	0.00
Population (ln)	−0.09	0.03	0.00

Notes

Estimates come from seemingly unrelated (OLS) regressions. Standard errors are not clustered. The unit of analysis is the individual project. The dependent variable is the project effectiveness score treated as a continuous variable. Project traits are controlled for. Donor and completion year fixed effects are used. The top panel shows the combined impact of all the mediators on the Pacific effect. The lower panel shows the impact of individual mediators.

The first portion of Table 5 shows the original Pacific effect (the negative impact of the Pacific on aid project effectiveness). It also shows the reduction in effect associated with the combined mediator values, and it shows the remaining Pacific effect. As in the analysis shown in Table 4, Table 5 shows that the mediators more than fully account for the Pacific effect. In other words, were it not for the traits we have studied, aid projects would possibly be slightly *more* effective in the Pacific than in the rest of the world.

The second portion of Table 5 is devoted to the individual mediating effects of each of the mediators. (The effect sizes in the table can be interpreted as the extent to which they change the coefficient for the Pacific). As in Table 4, governance’s effect is in the opposite direction. Were it not for better than average governance in the Pacific, aid projects would be less effective still in the region. Growth has a small, statistically significant, role in mediating the Pacific effect. The impact of GDP per capita is effectively zero. Freedom is a large part of the explanation as to why aid projects are less effective in the Pacific. Taken together, remoteness and population serve as larger constraints still: isolation and small population sizes appear to take a particularly heavy toll on project effectiveness in the Pacific.

There are three potential methodological shortcomings in the analysis in Table 5. The first is that standard errors are not clustered. The second is that the regressions are OLS with project effectiveness treated as a continuous variable. Treating the dependent variable as continuous is in line with much of the existing literature. However, in Appendix 1, results of KHB models that allow standard errors to be clustered and the dependent variable to be treated as ordinal are presented. Results are substantively very similar to those presented in Table 5. Appendix 1 also contains further robustness tests in the form of more parsimonious regression models. Once again results are substantively the same.

The third potential shortcoming is to do with changes in measures such as governance, GDP, and economic growth over project lifetimes. As discussed above, these measures come from projects’ start years to reduce the risk of reverse causality. However, rates of economic growth change considerably over time (this is less true with other measures such as GDP and governance, although some change does occur).^6^ For this reason, notwithstanding the risk of endogeneity, a sensible robustness test involves analysis with these variables taken from different periods. Accordingly, in online Appendix 1 we include an alternate model in which completion year growth is used, as well as a model in which growth, GDP, and governance are averaged over projects’ lifetimes. Results in these models are broadly similar to those presented in the body of the text, although growth’s potential role as a mediator increases somewhat. The fact that the central findings do not change much is reassuring, although—as we note further in discussion—our solution to the problem of variables such as growth is imperfect and leaves further scope for future work.

In the final section of this article, with a view to aid practice, we examine the extent to which available project traits are associated with better or worse aid project effectiveness in the Pacific compared to the rest of the developing world. The purpose of this work is not to explain the Pacific effect. Rather, it is to show donors which project choices may be potentially problematic in the region. The number of traits we could study is limited, owing to limited available information comparable across donors. However, we were able to test whether project size and duration have a different impact in the Pacific. We were also able to compare whether effectiveness differs in different sectors when the Pacific is compared with the rest of the developing world. Results are shown in Table 6.

**TABLE 6:** Regression with interactions

	Basic	Country controls
In Pacific	−0.15	−0.24
	(0.73)	(0.74)
Sector (economic omitted)		
Education	0.05	0.07*
	(0.04)	(0.04)
Environment/water	−0.06	−0.09**
	(0.04)	(0.04)
Governance	−0.19***	−0.15***
	(0.04)	(0.04)
Health/population	−0.01	0.02
	(0.04)	(0.04)
Humanitarian	0.30***	0.36***
	(0.06)	(0.06)
Other	0.04	0.06
	(0.04)	(0.04)
Duration of project (days)	−0.00**	−0.00***
	(0.00)	(0.00)
Project size (USD natural log)	0.08***	0.06***
	(0.01)	(0.01)
Pacific # Education	−0.18	−0.20
	(0.16)	(0.16)
Pacific # Environment/water	0.02	0.02
	(0.26)	(0.24)
Pacific # Governance	−0.01	−0.04
	(0.16)	(0.16)
Pacific # Health/population	−0.24	−0.26
	(0.21)	(0.22)
Pacific # Humanitarian	−0.55**	−0.62***
	(0.22)	(0.22)
Pacific # Other	0.14	0.13
	(0.29)	(0.29)
Pacific # duration of project	−0.00	−0.00
	(0.00)	(0.00)
Pacific # project size	0.01	0.03
	(0.04)	(0.05)
Remoteness		−0.04***
		(0.01)
Population (ln)		0.02***
		(0.01)
Growth		0.01**
		(0.00)
GDP per capita (ln)		0.02
		(0.02)
Governance		0.29***
		(0.04)
Freedom		−0.03***
		(0.01)
Donor FE	Yes	Yes
Completion FE	Yes	Yes
R2	0.12	0.14
N	8062	8062

Notes

Estimates from OLS regressions with clustered standard errors in parentheses. The dependent variable is the project effectiveness score. Donor and completion year fixed effects are included in all models.

Two sets of regression results are presented in Table 6: one in which regressions are run without country‐level variables as controls, and one in which country‐level variables are added as controls. The interaction terms in the models demonstrate whether the project traits in the models are associated with different levels of effectiveness in the Pacific and elsewhere.

**FIGURE 2:**
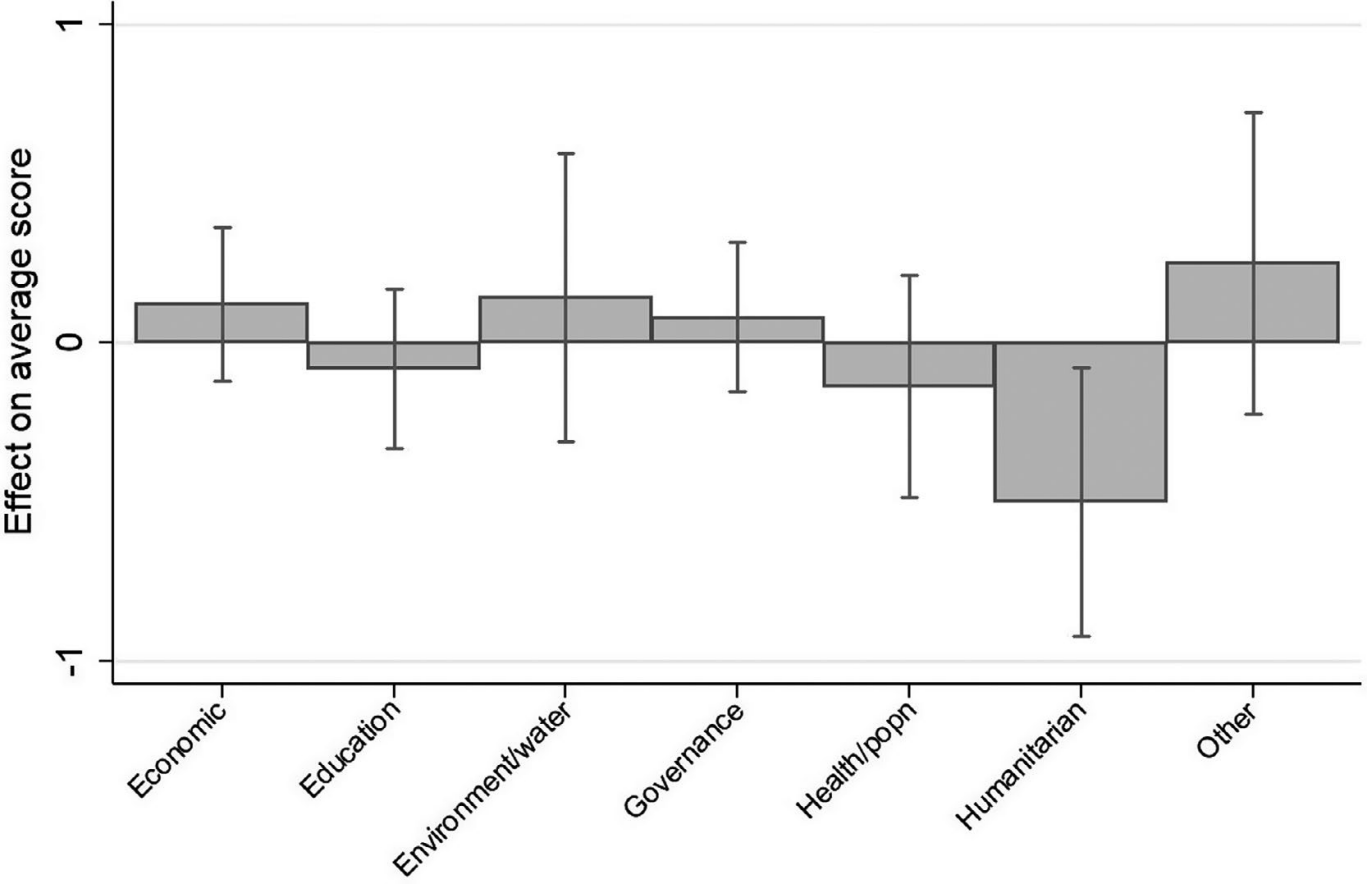
Differing sectoral performance in the Pacific compared with elsewhere *Notes*: The figure shows the predicted marginal effect of the difference in average performance between the Pacific and elsewhere for each sector. Estimates stem from the regression results shown in Table 6.

Although project duration and size have some impact on project effectiveness more generally, neither appears to have a differing impact on project effectiveness in the Pacific compared to the rest of the developing world. Indeed, the only variable for which any of the interaction terms is significant, is sector, and in particular humanitarian emergency work. For ease of interpretation, a margins plot showing the difference between the Pacific and elsewhere for all sectors is provided (Figure 2).

As the point estimates and confidence intervals show, no other sector’s performance differs between the Pacific and elsewhere in a manner that is statistically significant or in any way substantively meaningful. However, humanitarian projects do perform worse in a manner that is statistically significant. Questions can be raised about the substantive magnitude of this difference. It is less than one point on the six‐point scale used by donors when they appraise effectiveness. However, donor appraisals tend to cluster narrowly as donors are reluctant to award very high or low effectiveness scores to projects (Wood et al., 2020). As a result, the magnitude of differences such as that associated with humanitarian work in the Pacific may well be understated in regression models such as ours. As a result, the substantive magnitude of the finding is large enough to be of note. The finding is also important given the vulnerability of the Pacific to climate‐related emergencies such as tropical storms, as well as the risk posed to some Pacific countries by earthquakes, volcanoes, and tsunamis. Given remoteness and the challenges posed by geography, it is easy to imagine why the Pacific would be a challenging region for humanitarian responses. Nevertheless, the Pacific is clearly a region where humanitarian work needs to be as effective as possible.

One note needs to be added to the findings of this section. Multi‐collinearity was high in the regressions with interaction terms. While this cannot be a source of the finding related to humanitarian projects, it could plausibly be a source of the absence of findings associated with project size or duration. In an attempt to tackle issues of collinearity we reran regressions without completion year fixed effects. This reduced collinearity substantially but did not change results for size or duration.^7^

## 5 DISCUSSION

Two aspects of these findings may come as a surprise to researchers and practitioners with experience of aid in the Pacific. First, that the countries of the Pacific are comparatively well governed and that this increases aid project effectiveness. And, second, that comparatively high civil and political freedoms in Pacific countries appear to reduce aid project effectiveness.

Both findings are empirically consistent. Better governance is associated with greater effectiveness in much of the global literature on aid project effectiveness, and governance is better, on average, in the Pacific. Similarly, freedom has been found to be associated with worse aid outcomes in a number of global studies, and political and civil liberties are greater, on average, in the Pacific. Yet, influential scholars of the Pacific have tended to emphasize civil and political liberties in the region is a strength (Reilly, 2006), and poor governance as an impediment to development (Hughes, 2003).

In the case of governance, it is worth emphasizing what our findings do not show: they are not evidence that governance is not an impediment to aid project effectiveness in the Pacific. Rather, they are evidence that *compared to the rest of the world* governance is not a particularly acute constraint on project effectiveness in the region. At the same time though, our findings should serve to nuance public debate about aid in the region. Talk of governance in the Pacific is often dominated by problems and parts of the region where governance is at its worst. Inadequate attention is paid in scholarly work on aid and the Pacific to instances where governance is comparatively good. As a result, governance throughout the Pacific has often been viewed as a weakness by aid analysts (for the most influential example, see Hughes, 2003). Compared to Denmark this is true, but many Pacific countries are not particularly poorly governed by developing country standards, and certainly not so poorly governed as to render the effective delivery of aid impossible.

The freedom finding is consistent with other work on aid project effectiveness (for example, Feeny & Vuong, 2017), but it is hard to see why civil and political liberties themselves would be an impediment to aid success. Although we cannot be certain on the basis of analysis in this study, we think a likely explanation for the freedom finding is that this variable is tapping into something else—quite possibly the patronage‐oriented nature of politics in many Pacific democracies. Pacific countries are largely democratic, and liberties are not formally constrained, but a political culture of patronage is prominent in many Pacific democracies (Duncan & Hassall, 2011).

There is evidence from other aid studies that clientelist politics reduces aid effectiveness (Cruz & Keefer, 2015; Wright, 2010). There is also some evidence of politicians in developing countries being able to divert aid flows in politically advantageous ways (Briggs, 2014). Given this, it seems very plausible that the intersection of patronage and democracy in the Pacific may be the actual impediment to project effectiveness, rather than liberties as such.

This point speaks to an important area for building on our work in future research. Our study is the first to have focused on explaining problems of aid project effectiveness in the Pacific using quantitative methods. It is also the first study to have used causal mediation analysis in studying aid effectiveness at the project level.

Yet, while we view the subject matter of our research of considerable practical importance, and while we think our study has delivered clear insights, we do not believe our findings should be treated as the final word on the matter.

Empirically, we faced challenges. First—as with almost all other studies on aid project effectiveness—we struggled with the volatility of economic growth in developing countries and the potential endogeneity of this variable. We have tried, particularly in further work in the appendices, to mitigate risks associated with this problem. Our key findings for governance, freedom, population, and isolation did not change dramatically as we did this. For this reason, there are reasonable grounds to trust these results. Findings for growth did change somewhat, although it is unclear exactly how to interpret these changes owing to the risk of endogeneity. With more data in the future, it may be the case that more sophisticated methods can be brought to bear on this particular finding—existing uncertainties provide good grounds for further research.

What is more, as with all observational studies, including all existing work on aid project effectiveness, our findings could be biased by omitted variables. All of the variables we studied were included with a clear justification based on existing findings or other relevant work. We did not exclude any variables that had been found to be relevant in other work, which we could obtain data for, and which might plausibly explain the Pacific effect. We also added fixed effects and project‐level controls to our models. However, it may still be the case that key variables were missing from our analysis. These could include variables related to the nature of democratic politics—possibly, if a variable existed on the patronage nature of politics, and if it were included in our models, Freedom might cease to be a significant mediator. Testing the effects of these variables will be an important task for future work, although the absence of small island states from most potentially useful political datasets will pose a major challenge. It may well be the case that the most fruitful avenue of future study into challenges to aid effectiveness in the Pacific will involve different methods including qualitative methods such as Process Tracing.

There is also scope for additional work studying interactions between the Pacific and other project‐level variables. Our finding about the comparative ineffectiveness of humanitarian emergency aid projects in the Pacific is noteworthy. Such aid is important in the region and the need for it will likely rise. Donors need to work to try and increase the efficacy of this type of work.

While this finding is important, an acknowledged weakness of the second section of our analysis is the limited number of project traits available for us to compare between the Pacific region and other countries. There was a good reason for this limitation: donors do not make many more relevant variables available in a way that would allow for inclusion in a multi‐donor dataset. With persistence and donor cooperation, however, this stake of affairs could be changed. If it is, many more valuable insights about aid practice, and the relative importance of aspects of aid practice in different areas could be garnered (for an excellent example of this already occurring, see Honig, 2018).

Increased data availability could lend itself to other innovative forms of study. Although, on average, projects perform worse in particular parts of the world, such as the Pacific, and in more challenging country contexts, not all projects do. In future work, positive outliers, projects that overperform based on expectations could be identified from the residuals emerging from aid project effectiveness regressions, and then systematically studied using case studies (for an explanation of this method more generally, see Peiffer & Armytage, 2019).^8^ Positive outliers of this nature would best be identified in regressions run with good data on project traits. The case studies that emerged would similarly be at their most useful if rich information on the projects was available. Given this, the case for donors and researchers collaborating in this area is strong.

For the time being, there are still lessons for aid practice that can be drawn from our work. While it may seem like a counsel of despair to have found evidence that suggests the main impediments to aid effectiveness in the Pacific are either traits that cannot be changed (remoteness and population) or traits that we would not want to change (the presence of freedoms), useful takeaways can still be pointed to.

In particular, as the main constraints to effective aid are constraints that cannot be shifted or which should not be changed, donors ought to focus foremost on adapting their practice. Successful adaptation is not likely to involve changes in sectoral focus or project size or duration, but rather working in a manner appropriate to giving aid in difficult circumstances. Such a suggestion is itself not radical, there are already well‐known approaches to improved development practice such as Problem Driven Iterative Adaptation that emphasize the importance of contextually appropriate work and built‐in flexibility in projects (Andrews et al., 2013). Honig (2018) also provides compelling evidence that aid projects are most likely to be effective when practitioners are given freedom to adapt to circumstances.

Yet, it is not clear that such approaches have—thus far—permeated to significantly influenced donor practice in the Pacific. More investment in building donors’ own expertise in the region will also likely help, as will more investment in gold standard evaluations that allow donors to learn from the specific challenges confronting their work in the Pacific. As we have outlined, there is also scope for further partnership with researchers. Not all projects fail in the Pacific, and careful analysis combining both quantitative work and case studies has the potential to help inform donors about approaches that may work well.

Rates of development progress are low in much of the Pacific. The countries of the region will need aid for a long time to come. Aid can work in the Pacific, but making aid more effective in the region will be a challenge—one that in our view requires adaptation and further learning about the region’s context.

## Data Availability

The data that support the findings of this study are openly available in figshare at https://doi.org/10.6084/m9.figshare.16598894.v1. Full details on data sources are provided in the codebook available with the dataset.

## Footnotes

^1^
All figures come from the authors’ calculations derived from the referenced dataset. Aid dependency is calculated as ODA/recipient gross national income. Calculations are based on the mean of the five most recent years with data. If the most recent year alone is used, the facts stated here remain unchanged.

^2^
The full dataset is available online. It contains details on all data sources. The dataset can be found at: https://doi.org/10.6084/m9.figshare.16598894.

^3^
In the online appendices, we present results from alternate specifications of our central analysis, run as robustness tests, in which end year and average values are used for several of the key variables assessed on the basis of their start year values in the results presented in the main text.

^4^
The Pacific effect, it should be noted, does not only exist in our post‐1996 dataset; it can be found clearly in the full dataset of all years too.

^5^
A possible explanation for lower apparent project effectiveness in the Pacific could be differences in donor appraisal practice. It is possible that donors may be too ambitious when setting project objectives in the Pacific or too harsh when evaluating projects. However, we think it unlikely to be the main source of the difference. The Pacific effect can be found for each of the major donors in the region. This is despite individual donors presumably having different practices and institutional incentives. Also, the effect, as we note above, can be seen in country‐level data and the views of aid workers.

^6^
We are grateful to a reviewer for raising this potential issue.

^7^
Specifically, the mean variance inflation factor across variables in the full model was 51.15. Without year fixed effects it was 14.05.

^8^
We are very grateful to a reviewing editor for raising this useful potential approach.

## APPENDIX 1 Robustness tests

The following two tables present the results of mediation analysis using the KHB technique. The first reports on an OLS regression with clustered standard errors; the second reports on an ordered logistic regression with clustered standard errors. Because the ordered logistic regression reports results as logits, the coefficients appear to be of different magnitude. However, the size of the coefficients relative to each other is very similar to findings in our in other analysis. This is also true of sign and statistical significance.

**Table:**

**OLS (clustered standard errors)**		
Pacific effect		
Without mediators	−0.150	
With mediators	0.016	
Difference	−0.166	
**Effect of individual mediators**		
Mediator	Effect	Clustered SE
Growth	−0.020	0.010
GDP	−0.003	0.003
Remote	−0.098	0.027
Population	−0.092	0.034
Governance	0.154	0.027
Freedom	−0.106	0.023

*Notes*: estimates from KHB models. Standard errors are clustered. The unit of analysis is the individual project. The dependent variable is the project effectiveness score treated as a continuous variable. Project traits are controlled for. Donor and completion year fixed effects are used. The top panel shows the combined impact of all mediators on the Pacific effect. The lower panel shows the impact of individual mediators.

**Table:**

**Ordered logistic (clustered standard errors)**		
Pacific effect		
Without mediators	−0.359	
With mediators	0.010	
Difference	−0.369	
**Effect of individual mediators**		
Mediator	Effect	Clustered SE
Growth	−0.029	0.017
GDP	−0.011	0.007
Remote	−0.189	0.049
Population	−0.209	0.065
Governance	0.266	0.049
Freedom	−0.195	0.043

*Notes*: estimates from KHB models. Standard errors are clustered. The unit of analysis is the individual project. The dependent variable is the project effectiveness score treated as an ordinal variable. Project traits are controlled for. Donor and completion year fixed effects are used. The top panel shows the combined impact of all mediators on the Pacific effect. The lower panel shows the impact of individual mediators.

The following two tables present regression results from more parsimonious versions of the regressions shown in the body of the text. In the first, growth and GDP per capita are dropped from the regression model. In the second, project controls and completion year fixed effects are dropped.

**Table:**

**OLS—Growth and GDP dropped**		
Pacific effect		
Without mediators	−0.150	
With mediators	0.013	
Difference	−0.163	
**Effect of individual mediators**		
Mediator	Effect	Clustered SE
Remote	−0.114	0.025
Population	−0.104	0.034
Governance	0.167	0.027
Freedom	−0.113	0.023

*Notes*: estimates are from KHB models. Standard errors are clustered. The unit of analysis is the individual project. The dependent variable is the project effectiveness score treated as a continuous variable. Project traits are controlled for. Donor and completion year fixed effects are used. The top panel shows the combined impact of all mediators on the Pacific effect. The lower panel shows the impact of individual mediators.

**Table:**

**OLS—All project controls (except donor FE) dropped**		
Pacific effect		
Without mediators	−0.270	
With mediators	−0.003	
Difference	−0.267	
**Effect of individual mediators**		
Mediator	Effect	Clustered SE
Growth	−0.011	0.010
GDP	−0.008	0.005
Remote	−0.102	0.027
Population	−0.185	0.036
Governance	0.148	0.027
Freedom	−0.109	0.024

*Notes*: estimates are from KHB models. Standard errors are clustered. The unit of analysis is the individual project. The dependent variable is the project effectiveness score treated as a continuous variable. Project traits are controlled for. Donor and completion year fixed effects are used. The top panel shows the combined impact of all mediators on the Pacific effect. The lower panel shows the impact of individual mediators.

The following two tables report the results of the seemingly unrelated regression models that are the centrepiece of the mediation analysis in the body of the paper. Instead of using economic growth from the project’s start year, the first model reports results from analysis run when economic growth comes from the final year of the project. The second model reports on results in which economic growth is averaged over the lifespan of all projects. In addition, averaged values for GDP per capita and governance are also included in the second model.

**Table:**

**Mediation** **analysis with economic growth from the year of project completion**			
Total effect	−0.16		
Mediated effect	−0.15		
Remaining direct effect	0.00		
**Mediator**	**Coefficient**	**Std Err**	**p‐value**
Governance	0.15	0.02	0.00
Freedom	−0.10	0.02	0.00
Growth (at project completion)	−0.04	0.01	0.00
GDP (ln)	0.00	0.00	0.17
Remoteness	−0.10	0.02	0.00
Population (ln)	−0.07	0.03	0.02

*Notes*: estimates come from seemingly unrelated (OLS) regressions. Economic growth is taken from the project completion date; all other variables are measured as in Table 5 in the text. Standard errors are not clustered. The unit of analysis is the individual project. The dependent variable is the project effectiveness score treated as a continuous variable. Project traits are controlled for. Donor and completion year fixed effects are used. The top panel shows the combined impact of all the mediators on the Pacific effect. The lower panel shows the impact of individual mediators.

**Table:**

**Mediation** **analysis with growth, GDP, and governance averaged over project lifespan**			
Total effect	−0.22		
Mediated effect	−0.13		
Remaining direct effect	−0.09		
**Mediator**	**Coefficient**	**Std Err**	**p‐value**
Governance (averaged)	0.19	0.02	0.00
Freedom	−0.11	0.02	0.00
Growth (averaged)	−0.07	0.01	0.00
GDP (ln ‐ averaged)	0.00	0.00	0.10
Remoteness	−0.06	0.02	0.01
Population (ln)	−0.08	0.02	0.00

*Notes*: estimates come from seemingly unrelated (OLS) regressions. Growth, governance, and GDP are averaged over project lifespans; all other variables are measured as in Table 5 in the text. Standard errors are not clustered. The unit of analysis is the individual project. The dependent variable is the project effectiveness score treated as a continuous variable. Project traits are controlled for. Donor and completion year fixed effects are used. The top panel shows the combined impact of all the mediators on the Pacific effect. The lower panel shows the impact of individual mediators.
